# Knowledge, attitudes, and practices of primary healthcare practitioners in low- and middle-income countries: a scoping review on genetics

**DOI:** 10.1007/s12687-024-00721-y

**Published:** 2024-08-09

**Authors:** Sarah Walters, Colleen Aldous, Helen Malherbe

**Affiliations:** 1 School of Clinical Medicine, College of Health Sciences, University of KwaZulu-Natal, Durban, South Africa; 2 Director of Research and Epidemiology, Rare Diseases South Africa, NPC, Bryanston, Sandton, Gauteng South Africa; 3 Centre for Human Metabolomics, North-West University, Potchefstroom, South Africa

**Keywords:** Knowledge, Attitudes, Practices, Primary healthcare practitioner, Low- or middle-income countries (LMICs), Scoping review, Genetic education

## Abstract

Individualised treatment, including genetic services, calls for an increased role of primary healthcare practitioners (pHCPs) in diagnosing and caring for individuals with genetic conditions. PHCPs' genetics knowledge and practices must be current to ensure adequate care. A scoping review was conducted to explore peer-reviewed articles on the knowledge, attitudes, and practices (KAPs) of pHCPs concerning genetics, genetic testing, and genetic services. English-language human genetics/genomics articles published between January 1990 and April 2022 in low- and middle-income countries (LMICs) were included. Twenty-eight articles from 16 LMICs in five World Health Organisation (WHO)-defined regions met the inclusion criteria and showed a steady increase in publications, with varied contributions by region. The Eastern Mediterranean Region (EMR) contributed the most articles (*n* = 8), while the Western Pacific Region (WPR) had the least (*n* = 2). Brazil published the most articles (*n* = 6), while ten countries contributed one article each. Fifteen articles included knowledge, 19 included attitudes towards genetics, and eight included genetic practices. The findings indicate that pHCPs in LMICs lack knowledge of genetics and its applications despite their positive outlook towards genetic services. Barriers such as limited resources, financial constraints, and cultural or religious beliefs hinder access to genetic services. Enhancing pHCPs' genetics education is vital for improving care for those affected by genetic conditions. The scarcity of literature in LMICs emphasises the need for research on educational interventions to improve patient outcomes and family support.

**Supplementary Information:**

The online version contains supplementary material available at 10.1007/s12687-024-00721-y.

## Background

Since the initial human genome draft in 2000 (Lander et al. 2001), genetic service demand has grown, promising more personalised medicine (Collins et al. 2003). Primary healthcare practitioners (pHCPs), often the first contact for patients (Acheson et al. 2005), face a global push to integrate genetics into primary care. This necessitates partnerships and equips pHCPs with essential genetics knowledge for identifying conditions, conducting tests, and interpreting results (Burke 2004; Haga et al. 2019), which is crucial for effective patient guidance and care.

The clinical genetics field, encompassing rare to common diseases influenced by multiple genes, has advanced rapidly due to more accessible and affordable genomic testing and technologies, including next-generation sequencing (NGS). This evolution, from linked marker analysis to whole genome testing (WGS), has significantly expanded testing capabilities and options within just over a decade (Burke 2002).

Integrating genetics into clinical practice requires knowledge and awareness, yet there is a noted delay in adopting new technologies and evidence-based findings (McInerney et al. 2012). Many non-genetic healthcare professionals feel unprepared to engage with genetic testing, risk assessments, and interpreting results due to the complexity of genetic terminology (Mikat-Stevens et al. 2015). Given the rapid advancements in genetics, primary healthcare practitioners must update their knowledge continually.

LMICs face severe resource constraints impacting healthcare services. Specific health challenges include low life expectancy at birth, high infant and under-5 mortality rates, and poor educational and health outcomes due to inadequate access to quality healthcare (Puchalski Ritchie et al. 2016). Extensive work on genetics KAPs has been reported in high-income countries (HIC). However, equivalent studies are lacking in LMICs, particularly in Africa. In South Africa (SA), the familiarity and comfort of pHCPs with genetics and appropriate genetic testing are unknown.

Therefore, a scoping review was conducted to interrogate the published literature for pHCPs’ genetic KAPs in LMICs. The aim was to evaluate the scope of the literature and investigate the focus and type of work being conducted.

## Methodology

The scoping review was conducted initially in May 2019 and updated in April 2022, in Johannesburg, South Africa, guided by the Arksey and O’Malley framework (Arksey and O'malley 2005) and PRISMA-ScR guidelines (Tricco et al. 2018). The timeline was selected to include ten years before the publication of the human genome in 2001 (Lander et al. 2001) when genetics practice was introduced into mainstream healthcare.

### Identifying the research question

The research question was: What is available in the literature about primary healthcare professionals' knowledge, attitudes, and practices about genetics and genetic testing in LMICs?

### Identifying relevant studies: information sources and search strategy

Comprehensive literature searches were performed using three electronic databases (Web of Science, PubMed, and EBSCO), selected for their medical and educational papers coverage and accessibility via the online library at the University of KwaZulu Natal (UKZN).

A Boolean search string was developed: ("health* practitioner" OR "health* professional" OR "health* provider" OR doctor* OR specialist* OR consultant*) AND (Attitudes OR Knowledge OR educat* OR competenc*) AND (Genetic* OR Genomic* OR inherit* OR herit* OR congenital). Terminology to specify LMICs was not included in the Boolean string to maximise and capture the full scope of the literature. HIC-focused articles were excluded as a last step to ensure no LMICs were excluded.

### Study selection: eligibility criteria

**The inclusion** requirements for the study were:

- Original, peer-reviewed research.
- Surveys and questionnaires on the KAPs of HCPs related to human genetics and genomics;
- Full-text articles available in English and;
- Conducted in LMICs as defined by the World Bank (The World Bank Group 2021).

Excluded articles were:

- Unrelated to human genetics/genomics KAPs;
- Focused /performed in HICs excluded at the last step;
- Surveys involving only super-specialists (e.g., fetal medicine specialists, clinical geneticists/genetic counsellors);
- Focused on treatment, ethics, counselling perspectives, personal perspectives, or solely patient perceptions;
- For education purposes only;
- Grey literature, clinical audits, case studies, training programmes, policy articles, scale assessments or tool validation, and
- Unavailable in English or full-text versions.

### Study selection

Search results were imported into the reference manager software EndNote 20^TM^ (Clarivate 2021); duplicates and irrelevant articles were excluded. The second round of screening was undertaken independently by all three team members (SW, HM, and CA) based on title and abstract. Discrepancies were resolved via discussion. The third round of screening the full-text papers was undertaken by SW and HM, following pilot testing by the team on a random article set to determine the final inclusion/exclusion criteria. All articles focusing solely on HICs were excluded. References in the final articles were also screened for relevance.

### Charting the data: data abstraction and data synthesis

SW developed a data abstraction template to include the country of study, date, focus, methodology, and conditions studied. For a detailed data framework, see Supplementary File 1. The articles were imported into NVivo® (QSR International Pty Ltd 2020). In this qualitative data software tool, the full text was coded and analysed by SW and HM, creating a qualitative framework developed from identified themes.

## Results

**Fig. 1:**
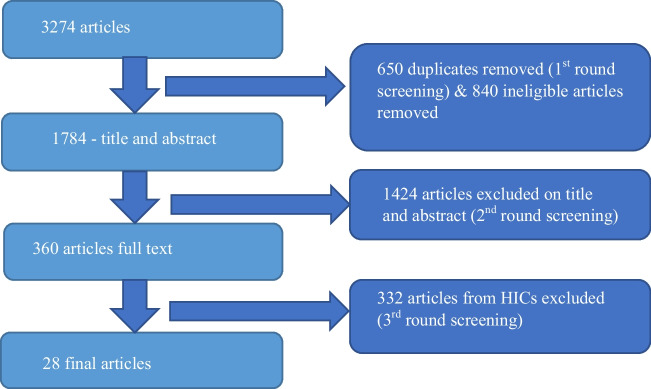
PRISMA Flow diagram of literature review and study selection process

A total of 3274 citations were identified. First-round screening excluded 650 duplicates and 840 irrelevant articles not meeting eligibility criteria. The second-round screening of 1784 articles was evaluated based on title and abstract, and 1424 articles were excluded. The third round of screening the full text of the remaining 360 articles excluded 332 articles (focusing on HICs), leaving 28 eligible articles for inclusion (Fig. 1 and Supplementary File 1).

### Study scope/characteristics

#### Geographical distribution of articles

The 28 included articles were published in 16 countries and across five WHO regions (Supplementary File 1 and Table 1). There were no articles from LMICs in the WHO-classified European region.

**Table 1:** Summary of the regions with the total number of LMICs and the number of published articles

WHO-defined Region	Total number of LMICs in the region	Number of countries that published articles and percent of total LMICs (%)	Number of published articles
EMR^1^	16	5 (31)	8
SEAR^2^	11	3 (27)	7
AMR^3^	25	2 (8)	7
AFR^4^	37	4 (11)	4
WPR^5^	23	2 (9)	2

Key: ^1^ Eastern Mediterranean region, ^2^ South East Asian region, ^3^ Region of the Americas, ^4^ Africa, ^5^ Western Pacific region

Brazil (AMR) published the most articles (*n* = 6), followed by Sri Lanka (SEAR) (*n* = 4), Lebanon (EMR), India (SEAR), Pakistan (EMR) and Iran (EMR), publishing two articles each. The remaining countries published one article each

### Timing of publications

**Fig. 2:**
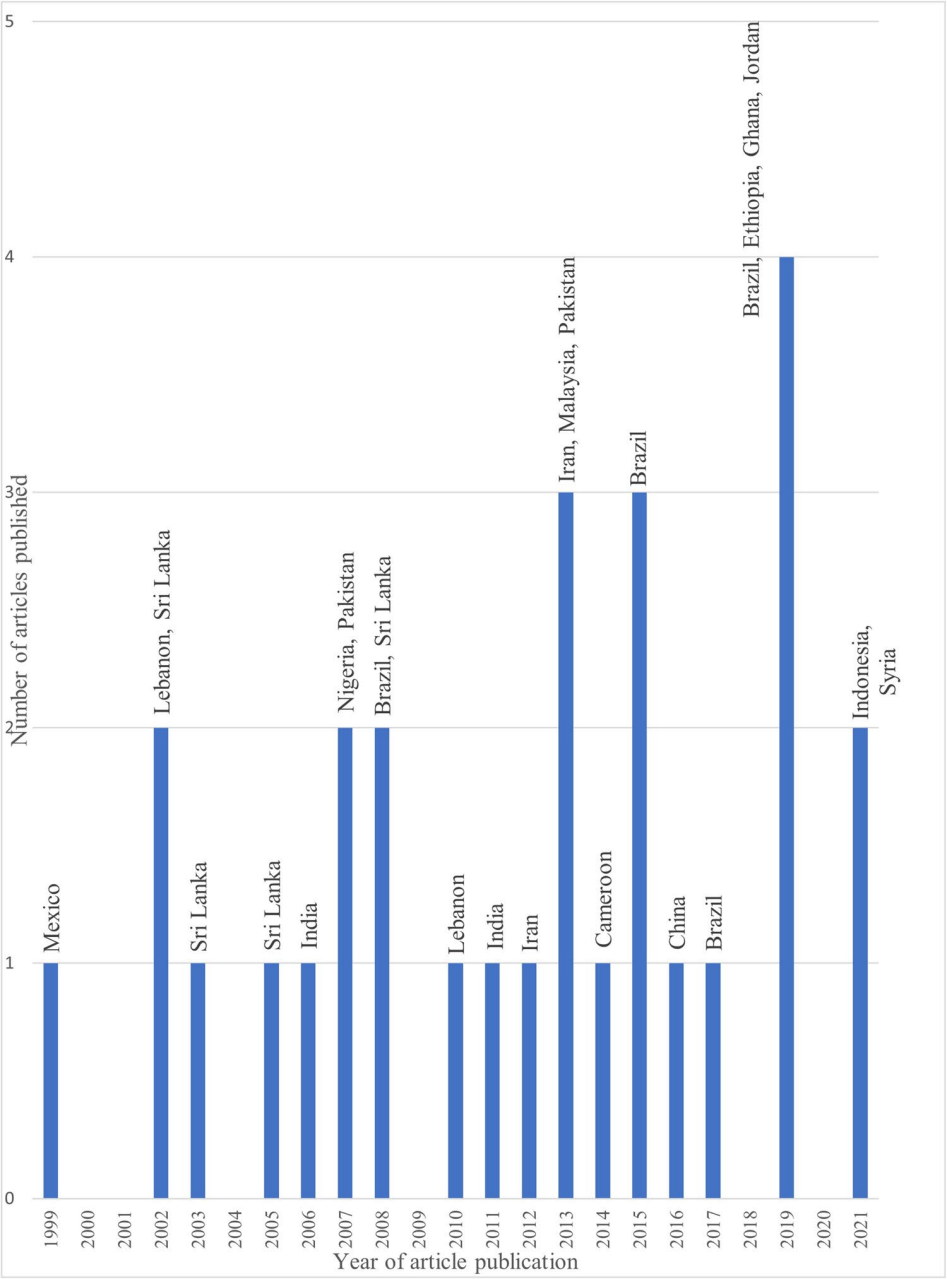
Article publication timeline including country of origin

**Fig. 3:**
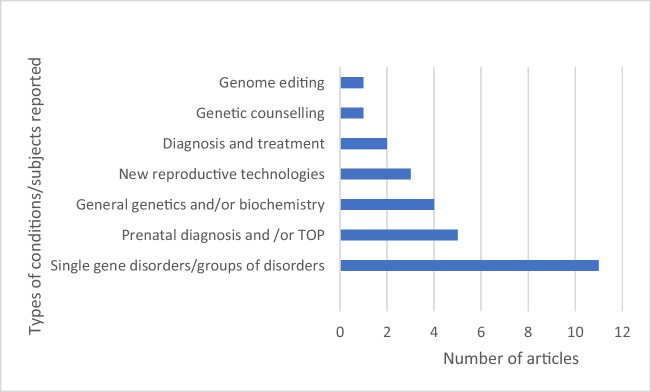
Types of genetic conditions/subjects surveyed in the articles

Mexico (AMR) published the earliest article in 1999. Nine articles were published from 2000–2009, 16 between 2010–2019 and two from 2021–2022 (Fig. 3). Articles from AFR first appeared in 2007 (Nigeria), and Malaysia (WPR) published their first article in 2013 (Fig. 2).

### Scope of genetics content

Eleven articles included research on one or more single gene disorders or groups of disorders caused by single gene pathogenic variants. The group of inherited blood disorders were the most frequently reported: sickle cell disease (SCD), thalassaemia, and other bleeding disorders (*n* = 6). SCD is featured in most articles in Africa (*n* = 3). Other single gene conditions included primary immune deficiencies (PIDs) (*n* = 2), Huntington’s disease (HD) (*n* = 3), and Brugada syndrome (*n* = 1).

The remaining articles focussed on broader genetic concepts. Prenatal diagnosis and termination of pregnancy (TOP) were grouped as one concept regarding reproduction (*n* = 5). General genetics and/or biochemistry as a teaching concept was included in four articles, and newer technologies (at the time), such as pharmacogenomics and non-invasive prenatal testing (NIPT), were discussed in three. Two articles broadly addressed the diagnosis and treatment of genetic conditions, and genetic counselling was the focus of one. Genome editing was reported in one article (Fig. 3).

### Study types

Twenty-seven studies used quantitative methodologies, some including a qualitative component, such as an interview tool (Iriart et al. 2019). Cross-sectional methodology was commonly used, using convenience sampling and a survey or questionnaire tool. For a detailed overview of the characteristics of the included articles, see Supplementary File 1.

### Emerging themes

**Fig. 4:**
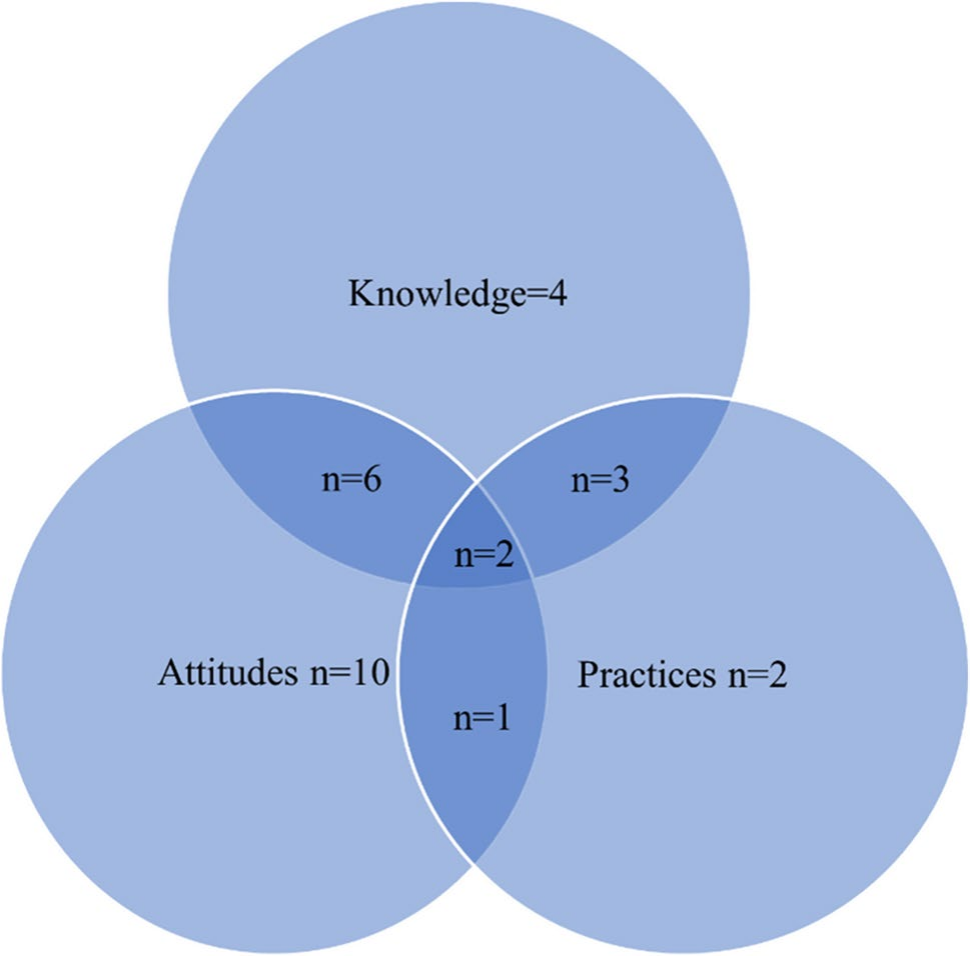
Coverage of the three topics assessed in the included articles (*n* = 28)

The articles included one, two or three aspects of healthcare workers' KAPs (Fig. 4). The majority focused on attitudes only (*n* = 10). Six focused on attitudes and knowledge, and one article focused on attitudes and practice.

**Fig. 5:**
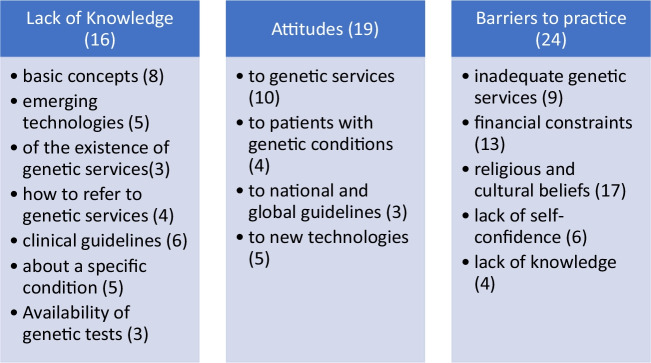
The thematic framework identifying the main themes in this scoping review; the number of articles per theme is denoted in brackets. Some articles were included in more than one theme

The qualitative thematic framework developed was consolidated under three headings: Lack of knowledge, Attitudes, and Barriers to practice, as defined by the research question (Fig. 5).

## Discussion

This review aimed to investigate the published literature on pHCPs' genetic KAPs of genetics and genetic services in LMICs. To our knowledge, this is the first review of this kind. Over the 32 years of study (1990–2022), 28 articles from 16 LMIC countries met the inclusion criteria.

The few relevant articles on HCPs’ KAPs may indicate the limited availability of genetic services in LMICs. HICs have been the forerunners in introducing and offering genetic testing for diagnosis and prenatal diagnosis, with research into genetics knowledge of pHCPs dating back to 1989 (Emery et al. 1999). In comparison, many LMICs lack comprehensive genetic services, reflecting the ongoing epidemiological transition in these countries – a process completed in most HICs decades ago (Malherbe et al. 2015).

### Geographical overview and topics investigated.

#### Eastern Mediterranean Region (EMR)

Research from this region included attitudes towards prenatal testing and TOP, haematological disorders, PIDs, pharmacogenomics and the provision of genetic services (Antoun et al. 2010; Ashfaq et al. 2013; Gilani et al. 2007; Nourijelyani et al. 2012; Robati and Farokhi 2013). A Jordanian study discussed attitudes toward genetics and biochemistry (Zahed et al. 2002). Many countries in the EMR, especially those with large Arab communities, exhibit higher consanguinity rates, which has led to an increased prevalence of congenital disorders (Dantas et al. 2015a, b). Surprisingly, this was only addressed in a few studies.

#### South-East Asia Region (SEAR)

A Sri Lankan HCP/research group published three articles on reproductive and genetic technologies, including TOP, from 2002 to 2005 (Dissanayake et al. 2002; Simpson et al. 2005; Simpson et al. 2003). They noted the unregulated emergence of IVF clinics in 1999. De Silva et al. (2008) examined TOP in Sri Lanka for specific genetic conditions: Down syndrome, haemophilia, spinal muscular atrophy I, and HD. Other studies explored late TOP for fetal anomalies (Phadke et al. 2011) and attitudes towards genome editing (Izzah et al. 2021). The scarcity of genetic education and resources in SEAR hinders the integration of genetic services, with ethical and equitable healthcare delivery being significant concerns exacerbated by socio-economic disparities (Rup et al. 2017). Despite WHO recommendations (World Health Assembly 2010), genetic services remain under-prioritised in LMICs, overshadowed by more immediate healthcare needs.

#### The Region of the Americas (AMR)

Brazil's early research included Brugada syndrome (Perez Riera et al. 2008) and articles from the Federal University of São Paulo on PIDs and non-communicable diseases (Dantas et al. 2015a, b; Ferreira et al. 2015; Melo et al. 2015). Genetic healthcare services were investigated (Iriart et al. 2019; Lopes-Junior et al. 2017), possibly prompted by the health system's response to the Zika outbreak (Pan American Health Organisation 2016). A 1999 study in Mexico investigated HD diagnostics (Alonso Vilatela et al.), an interesting topic in the evolution of genetic education, with genetics becoming mandatory in medical training only since 1996. Despite Mexico's estimated 5% prevalence of genetic disorders (Christianson et al. 2006), limited research exists on CDs (Dantas et al. 2015), reflecting a gap in genetics-related services and knowledge.

#### Africa (AFR)

Four articles in four countries of the 47 LMICs were included. Many African countries face a high burden of SCD. Three articles focused on pregnancy and SCD (Aboagye et al. 2019; Adeyemi and Adekanle 2007; Wonkam and Hurst 2014), the most common monogenic disorder in AFR (Rees et al. 2010) with a high birth prevalence in parts of sub-Saharan Africa (SSA) due to selection advantage with malaria (Piel et al. 2017). In Cameroon, where there is no universal health care or medical insurance, non-communicable diseases (NCD) such as SCD represent an increasing health burden (Wonkam and Hurst 2014), with SCD carrier rates estimated at 1 in 4 people (Adeyemi and Adekanle 2007). Although national SCD control programmes are partially implemented in these African countries, care for SCD-affected individuals is still lacking (Adeyemi and Adekanle 2007; Wonkam and Hurst 2014). Researchers have started assessing the integration of genetic services in primary healthcare, an ideal site for screening, antenatal care, and early childhood treatment of genetic conditions. Quinonez et al. (2019) investigated physicians’ genetics and genetic disease education in Ethiopia. The lack of capability due to personnel, expense, technology, infrastructure, and medical education was highlighted in implementing genetic services in LMICs equivalent to those in HICs. Successful strategies in HICs, such as prenatal diagnostic services, carrier screening programs, and newborn screening programmes, are lacking in many LMICs, leading to increased CDs.

#### Western Pacific Region

China and Malaysia published one article each (Ngim et al. 2013; Zhai et al. 2016), suggesting a lack of English language publications. Both articles discussed pregnancy and prenatal testing. The lack of NIPT coverage and affordability in China suggests that government funding would decrease the “societal burden of birth defects” and support national goals (Zhai et al. 2016). The availability of prenatal diagnosis and treatment for thalassemia at Malaysia’s public hospitals was investigated (Ngim et al. 2013). Despite progression, Malaysia lacks a formal rare disease definition, contributing to limited genetic healthcare resources (Shafie et al. 2020). An opportunity exists for pHCPs, government, and society to shape policy for genetics services that include epidemiological needs and appropriate resources (human, financial, structural) and include religious and social circumstances.

### Timeframe of publications

Within the study's time frame, a single article predates the year of the publication of the human genome (Alonso Vilatela et al. 1999). In the subsequent decade, nine studies were published, and an additional 18 were published from January 2010 to April 2022.

The ascending trend in the number of relevant publications originating from LMICs is encouraging. It suggests an increasing recognition of the need for clinical genetic services in the post-genomic era following the publication of the human genome. It may reflect the growing awareness and acceptance of the pivotal role of clinical genetic services in healthcare, particularly in LMICs. It is hoped that the medical curricula in LMICs' medical schools will continue to update their genetics content in the programme, enabling future pHCPs with improved genetic knowledge to provide genetic services for affected individuals and families.

#### Qualitative framework

The thematic framework (Fig. 5) was used to contextualise and evaluate the qualitative component of this review.

### Lack of genetic knowledge

Fifteen articles report a lack of genetic knowledge among pHCPs, affecting their ability to deliver quality genetic service and manage patients with genetic disorders (Suther and Goodson 2003). Self-rated assessments show knowledge ranged from very knowledgeable to insufficient, emphasising the need for continuous educational interventions (Aboagye et al. 2019; Alfaqih et al. 2019; Alonso Vilatela et al. 1999; Ashfaq et al. 2013; Dantas et al. 2015a, b; Ferreira et al. 2015; Iriart et al. 2019; Nourijelyani et al. 2012; Perez Riera, Filho, Uchida *receiving*okhi 2013) (Watson et al. 2001). Knowledgeable pHCPs reported receiving genetics education through additional graduate degrees, continuing medical education (CME) courses or board certification. Research shows that in the USA, Canada, Europe and HICs in Asia, there is a drive for better genetic education of non-genetics pHCPs, including service delivery (Chou et al. 2021; Talwar et al. 2017).

However, gaps remain, such as a lack of knowledge in prenatal screening and familiarity with relevant legislation such as the Medical TOP Act (Phadke et al. 2011). The lack of genetic training in countries like Ethiopia further hamper service delivery (Quinonez et al. 2019). Systematic reviews like that by and Tarini (2015) confirm that these educational and skill barriers compromise patient healthcare.

#### Lack of basic genetic concepts

Both pHCPs and nurses lack specific genetics knowledge, including prevention, prenatal screening, inheritance patterns, and informative family history collection (Alonso Vilatela et al. 1999; Ferreira et al. 2015; Melo et al. 2015; Quinonez et al. 2019). The needs assessment in Ethiopia (Quinonez et al. 2019) evaluated pHCPs’ experiences and proposed a way forward for genetic epidemiological studies. The results signify that well-integrated and supportive tools could enhance the effectiveness of genetic services by improving data collection and ultimately contributing to more informed decision-making and policy development in genetic services.

#### Clinical guidelines

Knowledge of and attitudes towards international, e.g., Center for Disease Control (CDC), national and local guidelines and protocols was poor. Ferreira et al. (2015) reported that only half of the pHCPs advised the use of folic acid for pregnant women, recommended as standard supplementation as far back as 1991 (MRC Vitamin Study Research Group 1991). Many LMICs now have folic acid fortification of foods, such as wheat or maize flour, but vary greatly in fortification legislation and implementation [Global Fortification Data Exchange. Map: Quantity and Proportion of Food Vehicle that is Fortified. http://www.fortificationdata.org Accessed 14/1/2024]. Only 10% of pHCPs reported knowledge of the international guidelines for genetic testing of Huntington’s disease (Alonso Vilatela et al. 1999). This deficit highlights the need for global education regarding guidelines and recommendations for CD treatment and care.

#### Emerging technologies

Many pHCPs are challenged with integrating current genetic knowledge into practice and risk being left behind in a rapidly evolving genomics era. Staying abreast of genetic advancements, including pharmacogenomics, WGS, whole genome association studies, and epigenetics, is challenging (Alfaqih et al. 2019; Dantas et al. 2015a, b; Izzah et al. 2021; Nourijelyani et al. 2012). Educational interventions, including continuing professional development (CPD) programmes, available online, through journals and conferences, and educational initiatives with access to genetic/genomic experts in other countries, are essential to update pHCP on the latest genetic developments, which could enrich their knowledge and contribute to patient care. Access, however, may be limited in LMICs, especially in under-resourced rural, poor, or sparsely populated areas.

Most pHCPs were reported to be optimistic about the impact of these emerging technologies (at the time of publication) on genetics and care. The role of genetics in designing targeted therapies in genetic variations which determine disease susceptibility was understood (Alfaqih et al. 2019). A similar attitude was noted with PGD for infertility (Dissanayake et al. 2002), implementation of NIPT in China (Zhai et al. 2016), and the future possibility of genome editing to treat fatal or debilitating diseases at both the embryonic and somatic levels in Indonesia (Izzah et al. 2021). Negative attitudes were expressed towards genome editing when it was applied to non-health-related aspects, i.e., eugenic uses (Izzah et al. 2021). The ethics of reproductive choices is intertwined with religious and moral belief systems, which differ within and between countries and individual pHCPs.

#### Lack of knowledge of specific conditions

PHCPs in the Middle East were found to underutilise genetic testing and services; the reasons needed to be clarified and explored, but lack of education, knowledge and expertise were thought to contribute (Antoun et al. 2010). Awareness and early identification of PIDs were more specifically reported (Nourijelyani et al. 2012). Lopes-Junior et al. (2017) concluded that pHCPs felt unprepared to provide genetic services in primary healthcare. Only 16% of pHCPs in Ethiopia reported that their genetics knowledge was sufficient for their practice, and most pHCPs were interested in further genetics education (Quinonez et al. 2019).

#### Existence of and referral to genetic services

The referral of patients in existing health systems in LMICs is challenging, as pHCPs lack awareness of existing genetic facilities, travelling distances, out-of-pocket expenses, and the time patients need to access these services. Chou et al. (2021) report on global barriers to accessing genetic services, identifying 12 HICs and one LMIC in their scoping review. While countries at all levels of development experience these challenges, in LMICs, this is compounded by other barriers and shortfalls in healthcare, exampled by pHCPs who are unaware of genetic services in their region and are less likely to order genetic tests, impacting healthcare quality for patients (Freedman et al. 2003). However, in LMICs, infrastructure, transport, sustainability, and funding remain persistent challenges to a greater degree (Olufadewa et al. 2021). Iriart et al. (2019) reported a lack of referrals from inland rural Brazil to the capital cities and urban centres, where genetic services are offered. Transportation challenges and travel costs in a resource-poor country are cited as significant obstacles for patients, resulting in a reluctance to refer by pHCPs. Strategies to expand and decentralise genetic healthcare may contribute to increased access to these services in LMICs.

#### Availability of genetic tests

Simpson et al. (2005) reported that although access to PGD in Sri Lanka was limited, many issues impacted HCPs' attitudes towards offering this. TOP is illegal in Sri Lanka unless the mother’s life is endangered. The authors commented on the “futility factor” of prenatal testing because of the continued illegality of TOP.

An interesting finding was that the availability of genetic services was not always related to their rate of use (Antoun et al. 2010). Middle Eastern countries have genetic testing and services available. Still, they are under-utilised, partially because pHCPs lack knowledge about genetic conditions, testing, and awareness of genetic services in their region. However, pHCPs still favoured referring to genetic services rather than counselling and requesting genetic testing, thus highlighting the importance of the availability of genetic services.

#### Attitudes of primary health care practitioners towards

##### Genetic services

Nineteen articles highlighted a consensus on the importance of genetic testing and counselling, with positive attitudes prominent among general practitioners (GPs) and pHCPs towards genetic services and integrating genetic counsellors into existing healthcare infrastructures to improve genetic services in general (Alfaqih et al. 2019; Alonso Vilatela et al. 1999; Ashfaq et al. 2013; Gilani et al. 2007; Melo et al. 2015). Moreover, supportive attitudes towards genetic counselling, prenatal diagnosis, and selective TOP contingent on TOP legislation were reported (Aboagye et al. 2019).

In contrast, Wonkam and Hurst (2014) emphasised that genetic services challenge the pHCPs’ "power to cure," possibly leaving the pHCPs feeling helpless and thus impacting their confidence in genetic services; PHCPs’ insecurity in prescribing medication for patients with genetic conditions was also expressed (Iriart et al. 2019). Despite treatment advances, the costs and access to new treatments in AFR for SCD remain prohibitive (Esoh et al. 2021). PHCPs face bureaucratic and funding challenges in accessing appropriate patient care (Iriart et al. 2019). They encounter difficulties such as emotional burden, prioritisation of infectious diseases, fears of discrimination and stigma, and stress, highlighting the need for their own education and emotional support (Gilani et al. 2007). A lack of care may also leave patients and families feeling further marginalised and stigmatised by the inaccessibility of appropriate treatment, enhanced by the reluctance of pHCPs to care for patients they cannot treat appropriately.

Alfaqih et al. (2019) reported on the diverse views around establishing a DNA database to enhance personalised medicine, and Albitar and Alchamat (2021) reported pHCPs’ uncertainty about requesting pharmacogenomics testing and for which medications, which links to lack of education and experience. Hopefully, as related knowledge increases and genetic test application improves, pHCPs' attitudes will improve.

##### National and global guidelines

Country-specific or international guidelines for the prevention and /or treatment of genetic conditions may be available. However, these were not adhered to either because of a lack of awareness or training. Ferreira et al. (2015) reported that pHCPs’ attitudes toward the implementation of either a municipal protocol or the CDC guidelines for the prevention of birth defects, especially in the pre-conception period, were non-compliant as they had not received training related to the guidelines. Nourijelyani et al. (2012) reported that the CDC had proposed strategies for the screening, identification, and surveillance of the PIDs. Still, training and re-evaluation of HCP attitudes are necessary to be effective. In India, HCPs expressed the need for a legal amendment of TOP for poor fetal outcomes to align with other countries (Phadke et al. 2011).

##### Barriers to genetic practices

Historically, barriers to developing and accessing genetic services have been reported (Suther and Goodson 2003). More recently, critical barriers to obtaining these services across both HICs and LMICs have been outlined as 1) Lack of knowledge and skills (the focus of this article); 2) Challenges in national healthcare systems; 3) Ethical, legal and social issues (ELSI), and; 4) Lack of an evidence base (Mikat-Stevens et al. 2015). Additional barriers include poor skills in taking family histories, non-existent referral guidelines and other tools, a lack of confidence in delivering genetic services, time constraints and the cost of tests (Antoun et al. 2010; Lopes-Junior et al. 2017; Melo et al. 2015; Ngim et al. 2013).

##### Religious and cultural beliefs

Diverse cultural and belief systems in LMICs may affect access to specific genetic services (Ashfaq et al. 2013). Religious beliefs were explicitly highlighted in two topics:

1. Termination of pregnancy
  Some religions do not permit TOP for foetal anomalies, and related laws and regulations vary within and between LMICs, limiting options for addressing affected pregnancies (Gilani et al. 2007; Ngim et al. 2013; Simpson et al. 2005; Simpson et al. 2003; Zahed et al. 2002). De Silva et al. (2008) reported that religious affiliation was the only variable influencing decisions regarding TOP. Even after amendments to relevant TOP legislation in countries such as Sri Lanka in 1995 (where TOP was strictly prohibited but is now permitted when the mother’s life is in danger), TOP for congenital disorders remains controversial, with religious leaders opposing this revised legislation (Dissanayake et al. 2002). The decreased uptake of this secondary prevention measure in both LMICs and HICs results in a higher affected birth rate, requiring lifelong care and considerable socio-economic impacts.
  Both pHCPs (De Silva et al. 2008) and patient perspectives (i.e. acceptance of fate and God’s will) were reported (Ashfaq et al. 2013). Religious affiliation was the common denominator regarding TOP decisions for affected pregnancies (De Silva et al. 2008). In contrast, the greater acceptance of TOP by pHCPs may be attributed to their awareness of the significant financial and social burden of caring for an affected child.
2. Emerging Technologies
  Reservations towards the use of some specific genetic technologies, e.g., PGD, gene therapy and gene editing, were noted amongst pHCPs. However, religious beliefs are not always a barrier to genetic services, as some doctors from a range of religious backgrounds support the newer genetic techniques to assist with reproductive difficulties (Dissanayake et al. 2002). As the education of pHCPs and the public improves around these issues, there may be a greater willingness to consider the benefits of these technologies.

### Financial constraints

#### Inadequate funding/ out of pocket expenses

Seven articles reported the barrier of inadequate financial resources allocated for genetic testing in LMICs (Aboagye et al. 2019; Antoun et al. 2010; Ashfaq et al. 2013; Dantas et al. 2015a, b; Lopes-Junior et al. 2017; Zahed et al. 2002; Zhai et al. 2016). LMICs cannot typically deliver genetic services on par with those offered in HICs (Quinonez et al. 2019). Essential genetic services are often viewed as superfluous expenditures in LMICs, and pHCPs frequently deprioritise CDs, particularly those considered untreatable (Lopes-Junior et al. 2017; Simpson et al. 2005).

These perspectives and fund allocation to more evident and "treatable" infectious diseases lead to insufficient budgeting for genetic services. Care for individuals with rare genetic conditions varies from only requiring dietary adjustments to inaccessible, prohibitively expensive treatment (Iriart et al. 2019). Most state-funded healthcare services in LMICs cannot offer treatment for rare diseases (Iriart et al. 2019; Simpson et al. 2005). Rare diseases are defined as a medical condition with a specific pattern of clinical signs, symptoms and findings that affects fewer than or equal to 1 in 2000 persons living in any WHO-defined region of the world (Rare Diseases International. Operational Description of Rare Diseases: Rare Diseases International; 2022 [Available from: https://www.rarediseasesinternational.org/description-for-rd/.) In many LMICs, the financial burden of healthcare is placed on families due to the absence of comprehensive health insurance coverage (Wonkam and Hurst 2014).

In LMICs, the lack of state funding shifts the financial burden of genetic testing to patients and their families, with costs often being prohibitive (Ashfaq et al. 2013). Studies in countries like Ghana, Lebanon, Pakistan, Brazil, and China report the unaffordable nature of genetic services, including carrier and prenatal screening (Aboagye et al. 2019; Antoun et al. 2010; Ashfaq et al. 2013; Iriart et al. 2019; Zhai et al. 2016). The cost of SCD carrier testing is a barrier (Aboagye et al. 2019), and Lebanese pHCPs are reluctant to offer genetic counselling due to expenses (Antoun et al. 2010). In China, a significant portion of pHCPs doubted women would opt for NIPT if it required out-of-pocket payment (Zhai et al. 2016), though coverage varies by region and timely healthcare access (Hu et al., 2021). These financial obstacles, including indirect costs like transportation, childcare, and lost income, exacerbate the impact of non-communicable diseases in LMICs, leading to higher disease burdens (March of Dimes 2006).

#### Inadequate genetic services

Although the availability of formal genetic services still needs to be determined in many LMICs, Kaur et al. (2019) indicated that limited genetic services exist in LMICs. While anecdotal evidence suggests a lack of clinical genetic services, little empirical evidence supports this, particularly in Africa. The Society for the Advancement of Sciences in Africa (SASA) reported that clinical genetic services are either non-existent or rudimentary across Africa, except for South Africa, which has a formal training programme to build capacity (Kapalanga 2020).

Many LMICs lack the necessary personnel, technology, infrastructure, and capabilities to offer a comprehensive clinical genetics service (Quinonez et al. 2019; Wonkam and Hurst 2014). No genetic counselling services are provided by the healthcare system in Pakistan (Riaz et al. 2019), which is compounded by a lack of clinical and genetic specialists, thus preventing access to the necessary counselling to accompany a diagnosis (Ashfaq et al. 2013; Zhai et al. 2016).

Some LMICs, such as Ethiopia, have no clinical geneticists or genetic counsellors (Quinonez et al. 2021). Encouragingly, Abacan et al. (2019) report on several LMICs that have genetic services provided by genetic counsellors. The findings are summarised in Table 2.

**Table 2:** Summary of the number of genetic counsellors and availability per million of the country's population

Region	Country	No. of genetic counsellors	Genetic Counsellors per million of the population	Reference
LMICs:				
AMR	Brazil	332	1.6	(Bonilla et al. 2021)
AFR	Ethiopia	0	0	(Quinonez et al. 2019)
AMR	Cuba	900	82	(Abacan et al. 2019)
Europe	Romania	75	4	(Abacan et al. 2019)
WPR	Malaysia	5	0.2	(Abacan et al. 2019)
AMR	Mexico	0	0	(Bucio et al. 2019)
SEAR	Philippines	11	0.1	(Abacan et al. 2019)
AFR	SA	20	0.4	(Abacan et al. 2019)
HICs:				
Europe	UK	310	5	(Abacan et al. 2019)
AMR	USA	4000	12	(Abacan et al. 2019)
AMR	Canada	900	9	(Abacan et al. 2019)
WPR	Australia/New Zealand	220	7	(Abacan et al. 2019)

SA is the only country with clinical geneticists and genetic counsellors in AFR. However, the 2019 data shows only 14 clinical geneticists (0.2 per million) and 20 genetic counsellors (0.4 per million), resulting in a capacity which remains far below the 120 clinical geneticists and 320 genetic counsellors recommended in 2001 (Department of Health 2001). Capacity is severely lacking compared with HICs such as the UK and USA and LMICs such as Cuba. Unfortunately, other LMICs, such as Mexico, have yet to recognise genetic counselling as a profession (Bucio et al. 2019). To increase genetic service capacity, several alternative, innovative options have been proposed by Mikat-Stevens et al. (2015), including telehealth and virtual consultations. The recent COVID-19 global pandemic has made virtual consultations a reality for many patients and pHCPs, including genetic services.

The challenges of limited resources, high local costs, and a lack of demand for genetic testing due to a continued focus on infectious diseases result in genetic tests being sent abroad. Many countries lack a database of locally available genetic tests, resulting in outsourcing genetic testing. Building local capacity remains an uphill struggle for LMICs when international companies offer equivalent testing at a lower price. In response to this need, the African Society of Human Genetics (AfSHG) and the Human Heredity & Health in Africa (H3Africa) aim to create and build genetic capacity by facilitating genetic research laboratory training at all levels to encourage genetic services and contribute to patient care (www.afshg.org).

#### Political will

For genetic services to be implemented, legislation must first be developed using appropriately allocated resources. SA has governmental policies for genetic services for individuals affected by genetic disorders (Department of Health 2001, 2003, 2005, 2021), but implementation is lacking. In Brazil, national policy has sanctioned the need to integrate genetic services into primary healthcare (Lopes-Júnior et al. 2014; Melo et al. 2015). This political commitment to genetic services is demonstrated by the number of medical geneticists in Brazil, as indicated above. In contrast, Wonkam and Hurst (2014) postulate that a high rate of termination of SCD-affected pregnancies suggests the failure of professional stakeholders to provide adequate care in Cameroon. It is hoped that Cameroonian research can develop capacity in the country in collaboration with international partners (Wonkam et al. 2009).

#### Lack of self-confidence

PHCPs reported feeling inadequate regarding certain aspects of genetic information, testing and particularly the construction and interpretation of family pedigrees (Lopes-Junior et al. (2017); Quinonez et al. (2019). PHCPs were also not confident in their ability to counsel or detect a genetic condition in a family. Mikat-Stevens et al. (2015) reported similar findings in HICs. Therefore, HCPs worldwide express the same opinions regarding the lack of knowledge and skills in genetics, which directly impacts patient care.

### Limitations of this study

This review was restricted to publications in English. Many LMICs are multi-lingual, with numerous languages and dialects within some countries, and relevant studies published in other languages may have been excluded. Additionally, the high-quality English required by many high-ranking, peer-reviewed journals may have prevented the publication of some LMIC manuscripts. Publication bias may be an issue, as positive outcomes of studies performed in LMICs may have a greater chance of being published than those with negative findings (Begg and Berlin 1989; Easterbrook et al. 1991). Additionally, adverse findings are not written up by the researchers. While this scoping review focused on non-genetic specialist pHCPs, some articles covered specialist and non-specialist audiences, which were retained to avoid omitting important information.

## Conclusion

Twenty years after the publication of the human genome, genetic knowledge and skills of pHCPs in South Africa and other LMICs remains limited. Providing at least the minimal genetic level of care for all affected patients and families while incorporating religious and cultural perspectives of different populations is crucial to avoid leaving patients without access to necessary, appropriate healthcare. Although many pHCPs are willing to incorporate genetics into their practice, the continued burden of infectious diseases, in parallel to a growing NCD burden, restricts their capacity. Expanding genetics knowledge and skills in these countries is also hampered by limited research to identify specific barriers to learning and implementing genetic services. Strategies to address these challenges, including genetic education from undergraduate to experienced pHCPs, are necessary, ensuring continuous updates in genetic knowledge and skills.

The way forward:

Based on the findings of this scoping review, recommendations to close this knowledge and skills deficit include:

- **Increased Governmental support**: To improve implementation of existing genetic and related policies in SA, practical workplans and approaches are needed to integrate relevant components of genetic services into primary healthcare, including maternal child and maternal health, and NCD care and management. This needs to be supported by evidenced-based allocation of human and financial resources.
- **Education and further training**: The genetics component of medical school curricula in LMIC requires review to ensure future HCPs are equipped with sufficient knowledge. This should be complemented by in-services training, as a component of continued professional development, to enable HCP to maintain their knowledge and skills in-line with emerging technologies. E-health technology could also be considered to enhance genetic services and education to understaffed healthcare facilities, particularly in rural and underserved areas.
- **Technology Infrastructure**: Laboratory equipment in genetic laboratories needs to be optimally maintained/serviced and updated to reflect emerging technologies and to ensure accurate diagnoses and improve research. Collaborative approaches should also be considered, such as the Nngwe project of the Diplomics initiative in SA.
- **Public awareness programmes**: Education of the public is also needed to create awareness and demand for genetic services. Appropriately pitched and innovative educational materials could be developed and made available in primary healthcare facilities in LMIC, ensuring consideration of cultural practices and traditional beliefs.
- **Screening programmes**: Implementation of newborn screening, carrier screening, preconception and prenatal screening would enable early identification of at-risk or affected people or pregnancies to ensure earlier care and interventions for better outcomes.
- **Improve clinical genetic counselling services**: An introduction of, or additional support for existing medical geneticists and genetic counsellors is required, to increase availability for HCP referral across the country, to assist families and individuals at-risk for or affected by genetic diseases.
- **Research**: Local research into common conditions in LMICs is essential to understanding the genetic health needs of the population. Research into the genetic diversity of a population will enable appropriate genetic testing and treatment. Regional collaborations would be beneficial for all populations in LMICs.

This review highlights the importance of further research on genetics in healthcare to better serve affected individuals and families, aligning with global health goals and to ensure that no one is left behind (World Health Assembly 2010).

## Supplementary Information

Below is the link to the electronic supplementary material.

Supplementary file1 (DOCX 23 KB)

## Data Availability

All data generated or analysed during this study are included in this published article and the supplementary information file. Use of Chat GPT OpenAI. (2024). *ChatGPT* (4) [Large language model]. https://chat.openai.com) and Grammarly (Grammarly Inc, 2009) were used for editing and information verification purposes only. Chat GPT was not used as a quotable source but served to enhance the clarity and coherence of the text. The authors reviewed the work thoroughly and take full responsibility for its content. The ethical and responsible use of AI was ensured throughout the process, adhering to established academic standards. No data or interpretations were altered by AI, and all intellectual contributions remain those of the authors. Proper oversight was maintained to ensure the integrity and originality of the research was preserved.

## Abbreviations

AFR: Africa Region
AfSHG: African Society of Human Genetics
AMR: Region of the Americas
CD: Congenital disorders
CDC: Center for Disease Control
COVID-19: Infectious disease caused by the SARS-CoV-2 virus
ELSI: Ethical, legal, and social issues
EMR: Eastern Mediterranean Region
GP: General practitioner
HCP: Healthcare practitioner
HIC: High-income countries
LMIC: Low- or middle-income countries
NGS: Next-generation sequencing
NIPT: Non-invasive prenatal testing
PGD: Pre-implantation genetic diagnosis
pHCP: Primary healthcare practitioner
SASA: Society for the Advancement of Sciences in Africa
SEAR: South East Asia Region
SSA: Sub-Saharan Africa
TOP: Termination of pregnancy
UKZN: University of KwaZulu Natal
WES: Whole exome sequencing
WGS: Whole genome sequencing
WHO: World Health Organisation
WPR: Western Pacific Region
