# Supplementary material for: Knowledge, attitudes, and practices of primary healthcare practitioners in low- and middle-income countries: a scoping review on genetics
Source: J Community Genet. 2024 Aug 9;15(5):461–74. doi: 10.1007/s12687-024-00721-y (PMC11549072; doi:10.1007/s12687-024-00721-y)
Supplement: Supplementary file 1 — Supplementary file1 (DOCX 23 KB) [file 12687_2024_721_MOESM1_ESM.docx]

**Knowledge, Attitudes, and Practices of Primary Healthcare Practitioners in Low- and Middle-Income Countries: A Scoping Review on Genetics**

**Journal of Community Genetics**

**Sarah Walters^1*^, Colleen Aldous^1^, Helen Malherbe^2,3^**

**^1^ School of Clinical Medicine, College of Health Sciences, University of KwaZulu-Natal, Durban, South Africa. ORCID: 0000-0003-4239-7897**

**^2^ Director of Research and Epidemiology, Rare Diseases South Africa, NPC, Bryanston, Gauteng.**

**^3^ Centre for Human Metabolomics, North-West University, South Africa**

**^*^Corresponding author:** [**216075854@stu.ukzn.ac.za**](mailto:216075854@stu.ukzn.ac.za)**, ORCID: 0000-0003-4239-7897**

**Supplementary Table 1: Final articles identified in scoping review.**

The scoping review was conducted using the Arksey and O’Malley framework (9) and in compliance with PRISMA-ScR guidelines. The location of the study per country and WHO region, and methodology was recorded. The participant knowledge, attitude and practices of genetics and genetic testing were noted to gain insight into which healthcare practitioners were interviewed/surveyed and for which genetic conditions. Articles are listed in alphabetical order of the country of origin.

| Year of study | Authors | Location | WHO Region | Aim of study | Methods | Participants | Knowledge | Attitude | Practice |
| --- | --- | --- | --- | --- | --- | --- | --- | --- | --- |
| 2008 | Perez Riera *et al*. | Brazil | Region of the Americas | knowledge of cardiologists regarding a low-prevalent entity associated with a high rate of sudden death—Brugada syndrome | Questionnaire | cardiologists | X |  |  |
| 2015 | Dantas *et al.* | Brazil | Region of the Americas | lack of awareness of PIDs among physicians | Cross-sectional, convenience sampling, with questionnaire | Paediatricians, internists, surgeons | X |  |  |
| 2015 | Ferreira *et al.* | Brazil | Region of the Americas | performance of doctors and nurses from a primary health-care unit in preventing birth defects in the preconception period based on the recommendations of the Control Center of Disease Prevention | Descriptive cross-sectional survey, semi-structured | Doctors and nurses | X | X |  |
| 2015 | Melo *et al*. | Brazil | Region of the Americas | genetic competencies of primary health care professionals | Descriptive survey | Doctors, nurses, dentists | X | X |  |
| 2017 | Lopes-Junior *et al*. | Brazil | Region of the Americas | Knowledge and experiences of genetics in primary healthcare | Survey | Doctors and nurses | X |  | X |
| 2019 | Iriart *et al.* | Brazil | Region of the Americas | therapeutic itineraries of patients and their families in search of diagnosis and treatment for rare genetic diseases in three regions, | Qualitative with semi-structured interviews | Endocrinologist, neurologist, biochemist, paediatrician, biologist, psychologist, nurse, nutritionist | X |  |  |
| 2014 | Wonkam & Hurst | Cameroon | Africa | attitudes regarding prenatal genetic diagnosis and termination of SCD affected pregnancy | Quantitative study with structured questionnaire | Doctors, parents, and adult patients |  | X |  |
| 2016 | Zhai *et al* | China | Western Pacific | willingness of Chinese obstetricians to offer NIPT and to determine how they would implement it and what resources they would need for the testing | Survey | Perinatalogist, obstetricians |  | X |  |
| 2019 | Quinonez *et al.* | Ethiopia | Africa | physicians’ education in genetics and genetic disease and their clinical experience and comfort with family history collection, diagnosis of a suspected genetic disorder, and delivery of genetic counselling | Survey | Physicians: paediatrics & Obstetrics/gynaecology |  |  | X |
| 2019 | Aboagye *et al.* | Ghana | Africa | educational practices of antenatal care providers toward pregnant women with sickle cell disease (SCD) and sickle cell trait (SCT) | Descriptive cross-sectional survey | Midwives, nurses, doctors at antenatal clinic | X |  | X |
| 2006 | Nagaraja *et al.* | India | South East Asia Region | attitudes towards presymptomatic testing for HD amongst HD family members, physicians, and laypersons | Questionnaire | doctors | X | X |  |
| 2011 | Phadke e*t al.* | India | South East Asia Region | opinion of lay persons and medical practitioners in India regarding late termination of pregnancies (LTOP) for fetal abnormalities | Questionnaires | Obstetrics/gynaecology, pathologists, microbiologists, surgery, anaesthetics, dermatology, paediatrics |  | X |  |
| 2021 | Izzah *et al.* | Indonesia | South East Asia Region | attitudes towards genome editing | cross-sectional study with online questionnaire | medical doctors and medical students |  | X |  |
| 2012 | Nourijelyani *et al.* | Iran | Eastern Mediterranean Region | knowledge of general practitioners and paediatricians about PIDs | Questionnaire | GPs and paediatricians | X |  | X |
| 2013 | Robati & Farokhi | Iran | Eastern Mediterranean Region | awareness of inherited bleeding disorders and anticoagulants | Descriptive analytical study with questionnaire | Dentists | X |  |  |
| 2019 | Alfaqih *et al.* | Jordan | South East Asia Region | attitude of physicians toward biochemistry and genetics including the correlation of their curricula with clinical practice | Cross-sectional study with questionnaire | Physicians | X | X | X |
| 2002 | Zahed *et al.* | Lebanon | Eastern Mediterranean Region | attitudes of health professionals in Lebanon towards prenatal diagnosis and termination of pregnancy | Questionnaire | Family doctors, paediatricians, obstetricians/ gynaecologist, geneticists |  | X |  |
| 2010 | Antoun *et al.* | Lebanon | Eastern Mediterranean Region | assesses use of and referral to clinical genetic testing and counselling, as well as the effect of education on their willingness to utilize or refer to these services | Questionnaire | GPs, family medicine, internal medicine, others |  | X |  |
| 2013 | Ngim *et al.* | Malaysia | Western Pacific | genetic counselling practices with regards to discussing prenatal diagnosis (PND) and termination of pregnancy (TOP) when counselling thalassemia carriers | Questionnaire | doctors and nurses |  |  | X |
| 1999 | Vilatela *et al.* | Mexico | Region of the Americas | knowledge and attitudes regarding the disease and its predictive and prenatal diagnosis of a group of Mexican specialists | Questionnaire | neurologists, psychiatrists, psychologists | X | X |  |
| 2007 | Adeyemi & Adekanle | Nigeria | Africa | Female healthcare worker awareness of pregnancy complications in sickle cell disease and how knowledge affects attitudes to early termination of an affected pregnancy detected by prenatal diagnosis | Questionnaire | Doctors, nurses, other healthcare workers | X | X |  |
| 2007 | Gilani *et al.* | Pakistan | Eastern Mediterranean Region | attitudes of doctors, medical students, lawyers, parliament members and parents of thalassemic children towards genetic diagnosis | Cross-sectional descriptive survey | Medical practitioners and medical students |  | X | X |
| 2013 | Ashfaq *et al.* | Pakistan | Eastern Mediterranean Region | views of medical doctors (MDs) towards genetic counselling services including what manner a master’s level genetic counselor might be incorporated into the healthcare system | Convenience sampling with questionnaire | GPs, paediatricians, ultrasonologists, obstetricians, gynaecologists | X | X |  |
| 2002 | Dissanayake *et al.* | Sri Lanka | South East Asia Region | attitudes towards various aspects of the new reproductive and genetic technologies | Questionnaire – pilot study | Doctors |  | X |  |
| 2003 | Simpson *et al.* | Sri Lanka | South East Asia Region | attitudes of doctors and medical students towards the new genetic and assisted reproductive technologies, issues of prenatal diagnosis and pregnancy termination | Survey | Doctors & medical students |  | X |  |
| 2005 | Simpson *et al.* | Sri Lanka | South East Asia Region | attitudes towards genetic counselling, amniocentesis and therapeutic abortion among doctors; focus on more recent developments in assisted reproduction such as IVF, Artiﬁcial Insemination by Donor (AID) and pre-natal genetic diagnosis (PGD), cloning and cryo-preservation | Survey | Obstetricians/gynaecologists, dentists, surgeons, administrators, physicians, ophthalmologists, family practitioners, paediatricians |  | X |  |
| 2008 | De Silva *et al.* | Sri Lanka | South East Asia Region | attitudes toward prenatal diagnosis (PND) and termination of pregnancy (TOP) for four conditions of differing severity | Questionnaires | Doctors, medical students, nurses, nursing students |  | X |  |
| 2021 | Albitar and Alchamat | Syria | Eastern Mediterranean Region | Knowledge of pharmacogenomics | Descriptive survey | pharmacists, physicians, others | X | X | X |
